# Th2A cells: The pathogenic players in allergic diseases

**DOI:** 10.3389/fimmu.2022.916778

**Published:** 2022-08-08

**Authors:** Ziyu Huang, Ming Chu, Xi Chen, Ziyuan Wang, Lin Jiang, Yinchao Ma, Yuedan Wang

**Affiliations:** ^1^ Department of Immunology, School of Basic Medical Sciences, Peking University, NHC Key Laboratory of Medical Immunology (Peking University), Beijing, China; ^2^ Department of Clinical Medicine, Mudanjiang Medical University, Mudanjiang, China

**Keywords:** allergic disease, Th2A cells, allergen-specific Th2 cells, HPGDS, CRTH2, CD161, ST2

## Abstract

Proallergic type 2 helper T (Th2A) cells are a subset of memory Th2 cells confined to atopic individuals, and they include all the allergen-specific Th2 cells. Recently, many studies have shown that Th2A cells characterized by CD3^+^ CD4^+^ HPGDS^+^ CRTH2^+^ CD161^high^ ST2^high^ CD49d^high^ CD27^low^ play a crucial role in allergic diseases, such as atopic dermatitis (AD), food allergy (FA), allergic rhinitis (AR), asthma, and eosinophilic esophagitis (EoE). In this review, we summarize the discovery, biomarkers, and biological properties of Th2A cells to gain new insights into the pathogenesis of allergic diseases.

## Introduction

Allergic diseases are the greatest prevalent chronic immunological diseases, including AD, asthma, and AR, which are estimated to affect more than 230 million, 330 million, and 400 million people worldwide, respectively (1–3). The prevalence of allergic diseases is increasing in both industrial and developing countries, making it a global epidemic (4). Notably, people with allergic diseases are more likely to have a family disposition known as atopy, defined by Coca and Cooke in 1923 (5). Many studies show that the progression of allergic diseases occurs in a predictable time sequence and is widely distributed to various organs, which is referred to as the atopic march (6). It is known that AD and FA in infancy gradually develop into asthma and AR in childhood, even EoE (7). However, the mechanism of the atopic march remains unclear.

It is known that allergic diseases are mainly driven by type 2 inflammation, mediated by Th2 cells (8). When atopic individuals are exposed to allergens, their epithelial cells start to secrete IL-25, IL-33, and TSLP, leading to the activation of dendritic cells, which promote the differentiation of allergen-specific Th2 cells (9, 10). The activated allergen-specific Th2 cells can not only favor B cells to produce allergen-specific IgE, but also recruit and activate basophils, mast cells, and eosinophils by secreting IL-4, IL-5, and IL-13 (11, 12). Thus, allergen-specific Th2 cells play a crucial role in the allergic response. Recently, pMHCII tetramer staining was widely used to study the allergen-specific Th2 cells (10, 13–17). However, the allergens in most allergic diseases are unclear. It is noted that Erik Wambre et al. defined a subset of memory Th2 cells confined to atopic individuals that include all allergen-specific Th2 cells as Th2A cells, which are characterized by CD4^+^ CRTH2^+^ CD161^high^ HPGDS^+^ CD27^low^ CD49d^high^ ST2^high^, providing a novel approach to study the allergen-specific Th2 cells (18). Here, we review the discovery, biomarkers, and biological properties of Th2A cells to gain new insights into the pathogenesis of allergic diseases.

## Discovery of Th2A cells

In type 2 inflammation, Th2 cells mainly trigger an inflammatory response, in which a subset of pathogenic effector Th2 cells (peTh2) are characterized by secreting high-level IL-5 cytokines (19, 20). After antigen elimination, most peTh2 cells are induced to undergo cell apoptosis, and part of them differentiate into memory-type Th2 cells (21). The memory-type Th2 cells in allergic diseases are activated by exposure to various allergens, which contribute to the recurrence of diseases. For instance, Kurokawa et al. demonstrate that the expression of ST2 in T cells is responsible for the pathogenesis of asthma (22). In addition, Calman Prussin et al. found that IL-5^+^ Th2 cells are highly differentiated Th2 cells that demand repetitive allergen exposure to generate, demonstrating that Th2-dominant diseases characterized by recurrent allergen stimulation possibly promote the generation of IL-5 Th2 cells, leading to the occurrence of eosinophilic inflammation (23). Moreover, Mitson et al. found that the CRTH2^+^ CD161^high^ HPGDS^+^ memory Th2 cells play a central role in AD and eosinophilic gastrointestinal disease (EGID) (20). Numerous studies demonstrate that the CD45RO^high^ CD69^high^ ST2^high^ IL17RB^high^ memory Th2 cells are the primary pathogenic subset in eosinophilic chronic rhinosinusitis (24–26). Meanwhile, Stephen Till et al. proposed that IL-17RB^+^ CD4^+^ Th2 cells may represent a subset that co-expresses ST2 during activation and may be pathogenic for chronic rhinosinusitis with nasal polyposis *via* the IL-25/IL-33 axis (27).

In 2012, Wambre et al. found the pathogenicity of allergen-specific CD4^+^ T cells to be associated with the CRTH2^+^ CD27^low^ phenotype (13). Wambre et al. proposed the Th2A cells, which can evaluate the severity of allergic diseases and the therapeutic effect of the allergen-specific immunotherapy (AIT) (28). In 2015, Wambre et al. clarified the importance of Th2A cells for allergic diseases and the possibility of Th2A cells as a target in AIT (29). Importantly, in 2017, Wambre et al. defined a subset of memory Th2 cells confined to atopic individuals that include all allergen-specific Th2 cells as Th2A cells, characterized by the expression of CD4^+^ CRTH2^+^ CD161^high^ HPGDS^+^ CD27^low^ CD49d^high^ ST2^high^ (18). Wambre et al. discovered that expression of a cardinal Th2 cytokine by peanut-reactive T cells was mainly restricted to allergic individuals and the Th2A cell subset and that a decrease in Th2A cells is also associated with peanut desensitization, confirming that Th2A cells are involved in the pathogenesis of FA (18, 30–32). Chiang et al. found the heterogeneity of effector Th2 subsets in food allergies (33). On the other hand, Renand et al. show that a decrease in grass pollen–specific CD4^+^ T cell frequency most closely paralleled the transient clinical outcome of allergen immunotherapy *via* either the subcutaneous or sublingual route (34). In 2020, Luce et al. found a decrease in Th2A cells after a period of food oral immunotherapy (OIT), but the frequency of DCs and ILCs remained, confirming the possibility of Th2A frequency as a new marker for OIT (35). In 2021, Bangert et al. found that Th2A cells were persistently maintained in the tissues of AD patients, suggesting that Th2A cells may be responsible for AD recurrence (36). Meanwhile, Luce et al. observed the frequency of CD38^+^ Th2A cells to be only significantly decreased in patients who received the treatment of HDM tablets, demonstrating the potential value of CD38^+^ Th2A cells as the new biomarker of asthma (37). In the meantime, Morgan et al. found that Th2A cells are enriched in esophageal tissue from EoE patients and associated with the recruitment of eosinophils to the esophageal tissue, indicating that Th2A cells are the pathogenic cells for EoE (38). A recent study by Vandamme et al. using TCR and transcriptomic analysis of dog allergen–specific T cell responses in allergic subjects revealed a close relationship between Th2-like, Th2, and Th2A cells (39).

## Biomarkers of Th2A cells

### HPGDS

**Figure 1:**
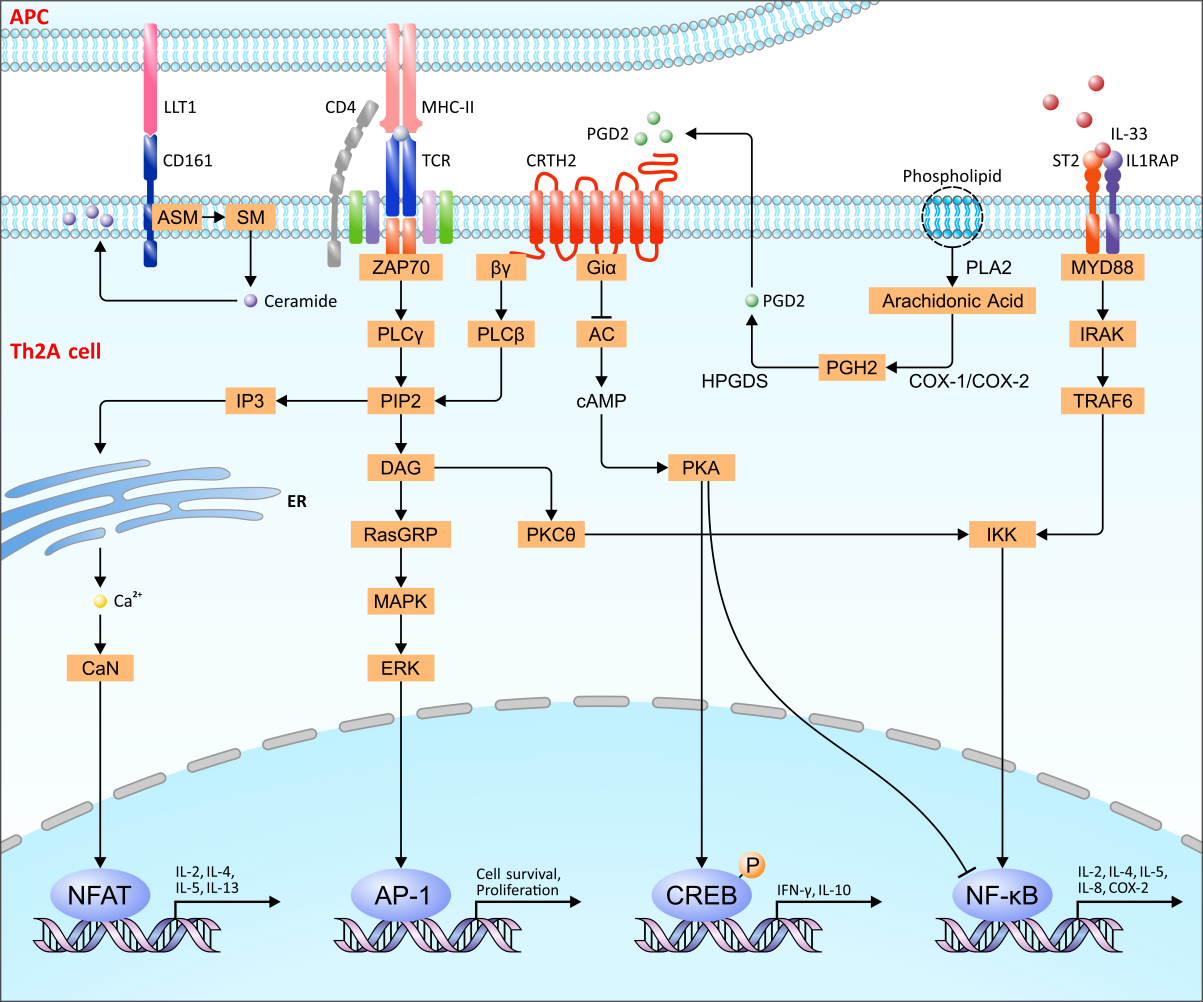
The signaling pathway of Th2A cells. The interaction of ST2 with IL-33 recruits MYD88 to activate TRAF6 by IRAK, leading to the activation of NF-kB released from the IKK complex. After CD161 combination with LLT1, aSMase is recruited to catalyze SM into ceramide, resulting in the aggregation in the membrane and inside the cells to mediate distinct functions. CRTH2 activated by PGD2 reduces cAMP production, thereby inhibiting NF-κB activity and phosphorylates CREB protein. Meanwhile, the same as TCR, CRTH2 also activates PKCθ, ERK/MAPK, and Ca2^+^ calcineurin signaling pathways to mediate cell survival, proliferation, and cytokine production through the transcription factor NF-κB, AP-1, and NFAT. Arachidonic acid derived from phospholipids is converted to PGH2 by COX-1/2, which can then be catalyzed into PGD2 by HPGDS. IKK, IκB kinase; ZAP70, ζ-chain associated protein kinase of 70 kDa; PLCγ, phospholipase Cγ; PIP2, phosphatidylinositol bisphosphate; DAG, diacylglycerol; PKCθ, protein kinase Cθ; RASGRP, RAS guanyl nucleotide–releasing protein; MAPK, mitogen-activated protein kinase; ERK, extracellular signal-regulated kinase; IP3, inositol trisphosphate; cAMP, cyclic adenosine 3, 5′-monophosphate; PKA, protein kinase A; COX-1/2, cyclooxygenases I and II; ASM, acid sphingomyelinase; CaN, calcineurin.

Unlike conventional Th2 cells, Th2A cells have a high expression of hematopoietic prostaglandin D2 synthase (HPGDS). HPGDS is a glutathione transferase expressed mainly in mast cells and antigen-presenting cells (40). Upon the stimulation of allergens, arachidonic acid is liberated from phospholipids under the mediation of phospholipase A2 (PLA2), and some arachidonic acid can be transformed into PGH2 by the action of COX (41). HPGDS then catalyzes the conversion of arachidonic acid–derivative PGH2 to PGD2 with the assistance of GSH and Mg2^+^ (42). PGD2 acts on the G-protein coupled receptor DP1 and receptor DP2/CRTH2 expressed in Th2 cells to promote inflammation by the secretion of IL-4, IL-5, and IL-13 cytokines (43) (**Figure 1**). Thus, the high-level expression of HPGDS in Th2A cells helps to promote the type 2 inflammatory response. The study shows that the HPGDS inhibitor improved AD symptoms, possibly due to a reduction in PGD2 production, indicating that HPGDS might be a potential therapeutic target for allergic diseases (44, 45).

### CRTH2

The chemoattractant receptor homologous molecule expressed on T-helper-type-2 cells (CRTH2) is a g-protein–coupled receptor of prostaglandin (PG) D2, mainly expressed in Th2 cells, eosinophils, basophils, and ILC2 (46–48). Many studies show that CRTH2 is one of the biomarkers of Th2A cells (18, 20). The combination of CRTH2 with PGD2 can induce the chemotaxis of Th2 cells, basophils, and eosinophils, recruiting circulating inflammatory cells from blood vessels to the site of inflammation (49–51). CRTH2 activated by PGD2 restrains adenylate cyclase (AC) activity through the Giα protein subunit, reducing the intracellular cAMP level, which can reduce the transcriptional activity of NF-κB and induce the phosphorylation of CREB protein to inhibit inflammation (52, 53). CRTH2 also activates the Gβγ complex to provoke the formation of PIP2 hydrolyzed by phospholipase Cβ, leading to the production of DAG and IP3 (52, 54). DAG can activate PKCθ and the MAPK/ERK pathway, activating the transcription factors NF-κB and AP-1, respectively (55). IP3 can induce the release of Ca2^+^ from the endoplasmic reticulum to activate transcription factor NFAT by the phosphatase calcineurin (52, 55) (**Figure 1**). As is known, CRTH2 is a significant marker of ILC2, which promotes the effector function in ILC2 (48). More importantly, the activation of CRTH2 stimulates Th2 cells to produce IL-4, IL-5, and IL-13 (56, 57). Moreover, CRTH2^+^ Th2 cells express more CD200R, which strongly correlates with Th2A pathology (58, 59).

### CD161

CD161, namely, NKR-P1, is a C-type lectin-like receptor mainly expressed in ILC2, NK cells, TH17 cells, and circulating memory T cells (48, 60). As the most significant biomarker in Th2A cells, CD161 is commonly regarded as the co-signaling receptor in T cells, the expression of which is related to memory phenotype (61). The binding of CD161 to LLT1 may recruit aSMase, which can upregulate Bcl-X_L_ expression by mediating IL-2 secretion, resulting in the reduction of susceptibility to apoptosis, leading to the prolonged survival of effector memory T cells (62, 63). More importantly, aSMase catalyzes sphingomyelin (SM) to generate ceramide (64) (**Figure 1**). Ceramide aggregates into a platform, which leads to the permeability alternation of the membrane (65–68). Intracellular ceramide affects the multifarious cellular processes and protein kinase activation (Ras/PKC), mediating the Akt-mTOR and MAPK cellular pathways downstream of CD28 and CD3 to regulate cell apoptosis, proliferation, and differentiation (62, 69, 70). Similar to Th2A, CD161 and CRTH2 co-expression are characteristic of ILC2 as well (48).

### ST2

Serum stimulation-2 (ST2), known as IL1RL1, is an orphan receptor mainly expressed in Th2 cells, Treg cells, Th9 cells, and ILC2s (71, 72). ST2 expression on peripheral allergen-specific CD41 T cells is confined to allergy individuals and restricted to Th2A cells and, on circulating CD4^+^ T cells, represents a transient phenotype associated with Th2A cell activation, allowing these cells to sense locally elicited tissue cytokines (73, 74). ST2 exists in two ways, which are different in signal transduction: a membrane-bound form and a soluble form (sST2) (71). After ST2 binds to its only ligand IL-33, the myeloid differentiation factor 88 (MyD88) is recruited to the intracellular domain, activating TRAF6 signaling *via* IL-1R-associated kinase (IRAK) (71, 75). Downstream MAPK kinases and the IKK complex activated by TRAF6 regulate the activation of AP-1 and the release of NF-κB from the complex, respectively (72) (**Figure 1**). However, sST2 inhibits IL-33/ST2 signaling by isolating free IL-33 (71). IL-33 selectively amplifies pathogenic Th2 cell effector functions, suggesting a tissue checkpoint that may regulate adaptive allergic immunity (73). Many studies show that ST2 in Th2A cells presents a transient phenotype related to cell activation, leading to the expression of IL-5 and IL-13 (71, 73, 76).

### PPAR

Peroxisome proliferator–activated receptor (PPAR) is a member of the nuclear receptor superfamily that is mainly expressed on macrophages and Th2A cells (77, 78). Activated PPAR forms a heterodimer with the retinoid X receptor (RXR), which can combine with the upstream PPAR response element to regulate the transcription of the target genes included in adipogenesis, lipid metabolism, inflammation, and metabolic homeostasis (79). PPARG can directly regulate the generation of IL-5 and the expression of ST2 both *in vitro* and *in vivo* (80). As mentioned, the IL-33/ST2 axis can strongly accelerate the Th2 cell function, indicating that PPARG might be a potential target for the treatment of allergic diseases (73, 80).

## Biological properties of Th2A cells

**Figure 2:**
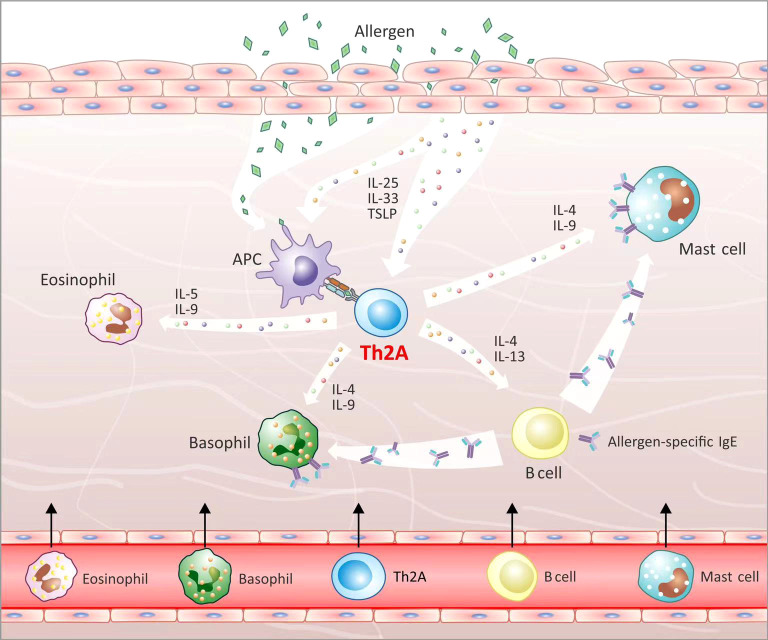
Th2A cells’ biological function. After allergen stimulation, epithelial cells secrete IL-25, IL-33, and TSLP to activate APCs and Th2A cells. APCs present allergens to activate tissue-resident Th2A cells. Th2A cells that have been activated secrete IL-5 and IL-9, which aid in the recruitment and activation of eosinophils. Th2A cells produce IL-4 and IL-13, stimulating B cells to produce allergen-specific IgE antibodies. Meanwhile, Th2A cells secrete IL-4, IL-9, and IL-10 to activate mast cells and basophils.

In the type 2 inflammatory response, epithelial cells and keratinocytes secrete IL-25, IL-33, and TSLP to activate ILC2, APCs, and Th2A cells, which induce the Th2 response to secrete IL-4, IL-5, IL-9, IL-10, and IL-13 in response to allergen stimulation (10, 11, 48). It is known that Th2A cells play a significant role in allergic diseases, but the mechanism remains unclear (18). We believe that, after exposure of atopic individuals to allergens, APCs present allergens to the tissue-resident Th2A cells, inducing cell activation (81). The activated Th2A cells may produce IL-5 and IL-9 to favor the recruitment and activation of eosinophils, which is supported by the fact that the Th2A subset presents a significant positive correlation with the number of eosinophils in the peripheral blood of atopic individuals (19, 20, 82). Meanwhile, Th2A cells may secrete IL-4 and IL-13, stimulating B cells to produce allergen-specific IgE antibodies that sensitize mast cells and basophils (11). In addition, Th2A cells may produce IL-4, IL-9, and IL-10 to activate the sensitized mast cells and basophils (11) (**Figure 2**). PGD2 secreted by the activated mast cells, in turn, acts on CRTH2 in Th2A cells, further promoting the activation of Th2A cells (83). Besides this, Th2A cells can survive in the tissues and blood for years, reserving the ability of allergen memory (73). Notably, ILC2s can induce tissue repair and result in the recruitment and activation of eosinophils and mast cells, which are functionally like Th2A cells and also essential to triggering the type 2 response but differ in that ILC2s have no TCR and allergen specificity (19, 84).

## Th2A cells in Allergic Diseases

### Th2A cells in AD

AD is the most common chronic inflammatory skin disease characterized by severe pruritus, recurrent eczema, and fluctuating course, which would gradually develop into FA, asthma, and even EoE (1). In 2021, Bangert et al. analyzed samples from the antecubital fossa of AD patients who had received the treatment of IL-4Rα blockers for 16 weeks and a year with single-cell RNA sequencing (scRNA-seq). Notably, a cluster of cells with IL17RB^+^ CRTH2^+^ CD161^+^ CD27^low^ phenotype most in accord with Th2A cells was found in the tissues of AD patients. Moreover, it is shown that even after a year of treatment, Th2A cells persistently existed in the patient tissues but were not present in healthy skin, indicating that Th2A cells are the main pathogenic subset for AD, possibly resulting in recurrent AD (36).

### Th2A cells in FA

FA is an adverse immune response induced by food allergens, including peanuts, milk, soy, and fish et al. (85, 86). Studies indicate that FA is caused by allergen-specific TH2 cells through the mediation of IgE switching and expansion of lymphocytes (87). Wambre et al. completed a longitudinal study on patients with peanut allergies who underwent characterized oral desensitization immunotherapy in 2017 (CODIT). In this randomized, double-blind, placebo-controlled experiment, most of the CD4^+^ T cells responding to peanuts were verified to be Th2A cells by a CD154 upregulation assay, which can observe the quantity of peanut-specific T cells *in vitro*. Furthermore, it is shown that a decrease in Th2A cell frequency is positively correlated with peanut desensitization, demonstrating the association of Th2A cells with the pathogenesis of FA (18). In 2020, Luce et al. recruited 60 desirable patients to be treated with omalizumab at weeks 1 to 16 and multi-OIT that included two to five kinds of allergens at weeks 8 to 30, and the patients received a food challenge at week 30. Fluorescent antibody labeling revealed a decrease in Th2A cell frequency at week 30 compared with the baseline. Inversely, no changes in ILC and DC frequency were observed through flow cytometry, indicating the value of Th2A frequency as a new marker for OIT (35). Meanwhile, Monian et al. subdivided the observed cells into six subsets through single-cell RNA-Seq and paired T cell receptor α/β sequencing, where inhibition of the Th2 signature in the Th2A subpopulation correlates with clinical outcomes of OIT (32). Moreover, Chiang et al. discovered that Tregs activated by IL-2 could partially inhibit the Th2 response, but the heterogeneous Th2 response was not significantly inhibited (33). Then, Lozano-Ojalvo found that IL-2 produced by allergen-specific cells can induce the activation of Treg cells with suppressive properties during an oral food challenge (88). In 2022, Bajzik et al. demonstrated that, during an experiment, the Th2A cell level observed in the peripheral blood of peanut-allergic patients is associated with not only T-cell reactivity to peanuts but also serum peanut-specific IgE and IgG4 levels (89).

### Th2A cells in Asthma

Asthma is a chronic allergic inflammatory disease characterized by multiple respiratory symptoms, airflow limitation, and reversible airway obstruction (2). A study was performed in 2021 by Luce et al. on 182 patients with allergies to house dust mites (HDM) to indicate the role of Th2A cells in allergic diseases. During the randomized, double-blind, placebo-controlled trial, these patients were treated with daily either 300 IR (*n* = 58), 500 IR (*n* = 63) HDM, or placebo (*n* = 61) tablets for 12 months. Flow cytometry was used to examine the changes in Th2A cell frequency between baseline and the end of AIT. Because both the active and placebo groups showed a decrease in Th2A cells, the same analysis was performed with CD38, which is upregulated in Th2A cells. The results suggest that a significant reduction in CD38^+^ Th2A was observed only in all active groups, confirming the potential value of CD38^+^ Th2A cells as the new clinical biomarker of asthma (37). Meanwhile, Blinova, EA et al. observed that, after treatment, the proportion of TH2A/Th2 cells decreased in the peripheral blood of asthma patients (90). Interestingly, in the ovalbumin-induced asthma mouse model, the expression of pathogenic Th2 cells in ST2^-/-^ and IL33^-/-^ mice is reduced significantly compared with ST2^+/+^ and IL33^+/+^ mice, resulting in observable improvement of pulmonary fibrosis, collagen deposition, and the prominent decrease in fibrosis-related gene expression, which indicates that ST2^hi^ memory pathogenic Th2 cells are involved in the establishment of airway fibrosis (91).

### Th2A cells in EoE

EoE is a chronic esophageal inflammatory disease characterized by pathological eosinophil infiltration, leading to dysphagia, food impaction, and impaired esophagus function (92, 93). In 2019, Rothenberg et al. utilized scRNA-seq to discover the specific enrichment of HPGDS^+^ CRTH2^+^ IL-17RB^+^ FFAR3^+^ CD4^+^ Th2 cell in EoE (94). In 2021, Morgan et al. collected biopsy tissue from the esophagus and duodenum of 10 patients with EoE (*n* = 6 active disease, *n* = 4 remission disease) and enzymatically hydrolyzed the patient tissues into a single-cell suspension for scRNA-seq. Th2A cells were prominently higher in the patients with active diseases due to the calculation of the two-sided Wilcoxon rank-sum test and enriched in the esophageal tissue of patients with EoE. In addition, the result of ligand-receptor pathway analysis shows that receptors selectively expressed on eosinophils are matched with the ligands on Th2A cells, indicating that Th2A cells may be related to the recruitment of eosinophils to the esophageal tissue (38).

## Immunotherapy targeting Th2A cells

It is known that, with insight into the immune mechanism, many targeted drugs are now applied to treat moderate and severe allergic diseases. As Th2A cells are pathogenic for allergic diseases, relative monoclonal antibodies are widely used, such as IL-4 monoclonal antibody (Pitrakinra, Dupilumab) (95–97), IL-5 monoclonal antibody (Mepolizumab, Reslizumab, Benralizumab) (98), IL-9 monoclonal antibody (MEDI-528) (99), IL-13 monoclonal antibody (Lebrikizumab, Tralokinumab) (100, 101), IL-33 monoclonal antibody (Etokimab, AMG 282) (102, 103), TSLP monoclonal antibody (Tezepelumab) (104), and IgE monoclonal antibody (Omalizumab, Ligelizumab) (105, 106). Importantly, JAK can regulate the full activation of Th2A cells, thus, JAK inhibitors also play an indispensable role in allergic disease treatment (107–114) (**Table 1**).

**Table 1:** Drugs targeting Th2A cells.

Target	Agent Name	Indications	Status	Reference
IL-4	Pitrakinra	asthma	Phase 2	(95)
	Dupilumab	AD, asthma, EoE,	FDA-approved	(96, 97)
IL-5	Mepolizumab	asthma	FDA-approved	(98)
	Reslizumab	asthma	FDA-approved	(98)
	Benralizumab	asthma, EoE	FDA-approved	(98)
IL-9	MEDI-528	asthma	Phase 2	(99)
IL-13	Lebrikizumab	AD, asthma	Phase 3	(100, 101)
	Tralokinumab	AD, asthma	Phase 3	(100)
IL-33	Etokimab (ANB020)	AD, asthma, peanut allergy	Phase 2	(102)
	AMG 282	chronic rhinosinusitis with nasal polyps, asthma	Phase 1	(103)
TSLP	Tezepelumab	asthma	Phase 3	(104)
IgE	Omalizumab	asthma	Phase 3	(105)
	Ligelizumab	asthma	Phase 3	(106)
JAK1	Upadacitinib	AD	Phase 3	(107)
	Abrocitinib	AD	FDA-approved	(108)
	Oclacitinib	Canine AD	FDA-approved	(109)
JAK1/2	Baricitinib	AD	FDA-approved	(110, 111)
JAK1/JAK2/JAK3/TYK2	Delgocitinib	AD	Phase 3	(112)
	Gusacitinib	AD	Phase 1	(113)

Moreover, Th2A cells have the possibility to be deleted selectively. Studies demonstrate that a persistent high dose of allergen cumulation in AIT might lead to the selective functional deletion of proallergic Th2 cells, which permits the occurrence of other T cell responses (29). Moreover, this chronic stimulation of high-dose allergens may induce autocrine IL-10 generation, which is the salient mediator of T cell exhaustion and the methylation of the GATA-3 promoter related to the decrease in Th2 cell activity (115, 116).

## Conclusion

Th2A cells are a subset of memory Th2 cells confined to atopic individuals, including all the allergen-specific Th2 cells. Notably, Th2A cells are characterized by the expression of CD4^+^ CRTH2^+^ CD161^high^ HPGDS^+^ CD27^low^ CD49d^high^ ST2^high^, which exhibit multiple biological properties leading to cell survival, activation, and cytokine secretion. After allergen exposure, APCs involve in allergen presentation to activate Th2A cells which can secrete IL-4, IL-5, IL-9, IL-10, and IL-13, leading to the activation of mast cells, basophils, and eosinophils. It is known that Th2A cells are demonstrated to be the central pathogenic cells in various allergic diseases, including AD, FA, asthma, and EoE. The frequency of Th2A cells can be used to assess the efficacy of allergic disease treatment, and selective Th2A cell deletion may result in long-term clinical benefits. Subsequently, Th2A cells are expected to be the focal point of research into the pathogenesis of allergic diseases and a critical target for disease treatment.

## Author contributions

ZH wrote most of the review. ZH and MC contributed to writing and correcting the manuscript. XC, ZW, LJ, and YM participated in critically correcting the manuscript. MC and YW contributed to the manuscript structure and supervised the work.

## Funding

Peking University Medicine Seed Fund for Interdisciplinary Research supported by “the Fundamental Research Funds for the Central Universities” (No. BMU2021MX021, No. BMU2022MX017). This work was supported by the National Natural Science Foundation of China (81603119) and the Natural Science Foundation of Beijing Municipality (7174316).

## Conflict of interest

The authors declare that the research was conducted in the absence of any commercial or financial relationships that could be construed as a potential conflict of interest.

## Publisher’s note

All claims expressed in this article are solely those of the authors and do not necessarily represent those of their affiliated organizations, or those of the publisher, the editors and the reviewers. Any product that may be evaluated in this article, or claim that may be made by its manufacturer, is not guaranteed or endorsed by the publisher.
